# Mogrosides Protect Against Diabetic Kidney Injury via Inhibiting Macrophage Activation in a Mouse Model

**DOI:** 10.1155/jdr/5291562

**Published:** 2025-06-24

**Authors:** Fangyi Jiang, Xiaoli Huang, Man Yan, Jiajun Tan, Xueyun Dong, Xianhai Liu, Jiayuan He, Asmaa Ali, Min Chen, Leilei Zhang, Liang Wu, Pingping Wang

**Affiliations:** ^1^Department of Laboratory Medicine, Gaoyou People's Hospital, Yangzhou, China; ^2^Department of Laboratory Medicine, Sihong Hospital, Suqian, China; ^3^Zhenjiang City Central Blood Station, Zhenjiang, China; ^4^Department of Laboratory Medicine, School of Medicine, Jiangsu University, Zhenjiang, China; ^5^Health Testing Center, Zhenjiang Center for Disease Control and Prevention, Zhenjiang, China; ^6^Department of Pulmonary Medicine, Abbassia Chest Hospital, EMOH, Cairo, Egypt; ^7^Public Experiment and Service Center, Jiangsu University, Zhenjiang, China; ^8^Department of Laboratory Medicine, Taizhou Second People's Hospital, Taizhou, China

**Keywords:** diabetic kidney disease (DKD), inflammation, mogrosides, NF-*κ*B/NLRP3/Caspase-1 axis, nontargeted metabolomics

## Abstract

Diabetic kidney injury is an almost unavoidable complication in diabetic patients. The activation of macrophages with high glucose in the patient's body is a key factor in triggering diabetic kidney disease (DKD). Mogrosides are commonly used sweeteners, but their effects on diabetic kidney injury are still unclear. This study used THP-1 cell models and diabetic mouse models to examine the impacts and pathways of mogrosides in inhibiting hyperglycemia-activated macrophages and alleviating kidney damage. This study used high glucose (33.3 mmol/L) to induce activation of macrophage-like THP-1 cells for studying the anti-inflammatory mechanism of mogrosides. At the same time, a diabetic mouse model was prepared using a high-fat diet and intraperitoneal injection of streptozotocin in order to further study the effects of mogrosides on alleviating symptoms of DKD. Mogrosides can suppress the activation of macrophages and kidney damage in diabetic mice, and this anti-inflammatory effect seems to be mediated through the NF-*κ*B/NLRP3/Caspase-1 axis in macrophages. Moreover, the metabolomic results revealed that the anti-inflammatory properties of mogrosides were associated with the modulation of glutamate metabolism and glycerophospholipid metabolism in macrophages. Our results indicated that supplementing diabetic patients with mogrosides may help inhibit inflammatory responses and prevent kidney damage.

## 1. Background

With the continuous development of the global economy and the increasing living standards of people, diabetes, as a chronic noncommunicable disease, has become a major global public health problem [1]. It is a serious endangerment to people's physical health and a serious impact on their quality of life [2].

Twenty percent to 40% of diabetic patients will develop kidney damage, which may further progress to end-stage renal disease [3]. Currently, diabetic kidney disease (DKD) stands as the primary contributor to end-stage renal disease, with poor prognosis for patients and no effective treatment methods yet [4]. The etiology of DKD exhibits intricate mechanisms, with oxidative stress and inflammatory reactions induced by high blood glucose being the main causes [5, 6]. In renal tissues of individuals with diabetes, multiple cell adhesion molecules, growth mediators, chemokines, and proinflammatory cytokines exhibit markedly elevated expression levels, subsequently triggering immune cells, including monocytes, lymphocytes, and macrophages, to contribute to the pathological injury mechanism of DKD [7, 8]. Macrophages are considered to be the main inflammatory cells involved in kidney damage, and animal model studies have confirmed that the number of activated macrophages is related to the severity of kidney damage [9, 10]. Stimulated macrophages generate substantial quantities of reactive oxygen species (ROS) to exacerbate kidney damage, and blocking macrophage activation is one of the effective strategies for treating DKD [11, 12].

Diabetic patients strictly limit the intake of sugars such as sucrose and fructose in order to control blood glucose levels within the normal range. In recent years, the emergence of various sweeteners has brought sweetness to the diet of diabetic patients, becoming essential in their lives [13, 14]. However, there is a lack of research on the impact of sweeteners on macrophages and kidney inflammation in diabetic patients. Mogrosides are sweeteners extracted from monk fruit, the main active ingredient of which is mogrosides, with a sweetness 200–350 times that of sucrose [15, 16]. Due to its high sweetness and nontoxicity, it has been widely added to various beverages and pastries [16]. Studies have shown that mogrosides have a wide range of pharmacological and health-promoting properties, including antitumor, anti-inflammatory, and antioxidant effects, but research on their impact on DKD is still lacking [17, 18]. This study used cell and animal models to investigate the effects of mogrosides on macrophage activation and DKD and explored the inhibitory effects of mogrosides on macrophage activation, as well as the anti-inflammatory and antioxidant stress mechanisms through cell metabolomics.

## 2. Methodologies and Materials

### 2.1. Cells and Experimental Groups

THP-1 cells were cultured using the method reported by Yan et al. and induced to differentiate into macrophages before the experiment [19]. In the cell culture medium, a final concentration of 33.3 mmol/L glucose (high glucose concentration) was added to trigger inflammation in THP-1 cells. The cell experimental groups included a normal control group (NC group), a high glucose group (H-Glu group), a high concentration mogroside group (H-Mo group), a medium concentration mogroside group (M-Mo group), and a low concentration mogroside group (L-Mo group). The treatment method for the H-Glu group involved incubating THP-1 cells in cell culture medium containing 33.3 mmol/L glucose for 12 h. In this study, high-purity mogroside V (purity greater than 98%) was provided by Nakeli-Biotech Co., Ltd. (Chengdu, China). Zhang et al. reported that incubating THP-1 cells with a final concentration of mogrosides V not exceeding 100 *μ*g/mL for no more than 48 h would not cause significant toxicity [20]. In this study, the final concentrations of mogrosides V in the H-Mo group, M-Mo group, and L-Mo group cell culture medium were 100, 50, and 25 *μ*g/mL, respectively. All three groups of cell culture media contained 33.3 mmol/L glucose and the corresponding concentrations of mogrosides V. The experiment was terminated after incubating THP-1 cells for 12 h. Upon experiment completion, the culture medium was drawn off, and the cells underwent three sequential washes with sterile phosphate-buffered saline to remove residual cell culture medium. The cell culture supernatant was collected for subsequent experiments.

### 2.2. Construction of Diabetic Mouse Models and Experimental Grouping

ICR mice (22 ± 4 g) were procured from Jiangsu Wukong Biotechnology Co., Ltd. (Nanjing, China) and raised in Animal Experiment Center of Jiangsu University. Animal experiments were performed in this study were approved by the Ethics Committee of the Jiangsu University (Protocol Code UJS-IACUC-AP-2022022804). The 18 mice were randomly divided into the NC group, the diabetes mellitus group (DM group), and the mogroside treatment group (Mo group).

The Yan et al. method was used to construct a diabetic mouse model [19]. The NC group mice received a standard diet, while the DM and Mo group mice were fed a 40% high-fat diet (Future Biotech Co., Ltd., Beijing, China). The ingredients of the high-fat diet can be found in Table S1. The mice received the high-fat diet across 4 weeks, and on the first day of the fifth week, they were induced with streptozotocin (STZ, 100 mg/kg) by intraperitoneal injection to induce diabetes. Before the end of the fifth week, fasting blood glucose (FBG) levels were measured in the mice, with a level greater than 11.1 mmol/L used as the criterion for successful construction of the diabetes model. Starting from the sixth week, the Mo group mice were given oral mogrosides treatment (mogrosides V > 98%, 50 mg/kg/day), with the dosage referenced from Liu et al. [21]. The above procedures were terminated at the completion of the 12th week.

After experiment completion, the mice underwent euthanasia through urethane administration (Sigma-Aldrich, United States, 700 mg/kg) via the intraperitoneal route, succeeded by the collection of blood and kidney specimens. Serum FBG in mice was measured by Siemens ADVIA Chemistry XPT System (Siemens Healthcare Diagnostics Inc. 5.11 Benedict Avenue Tarrytown, United States) in the Department of Laboratory Medicine of Gaoyou People's Hospital. Serum advanced glycation end products (AGEs) levels in mice were determined by a commercial kit obtained from Meimian Biotechnology Co. Ltd. (Yancheng, China).

### 2.3. qPCR Assay and Western Blotting Assay

Upon the completion of the experiment, the kidney tissue and the THP-1 cells were collected and washed twice with sterile phosphate-buffered saline. The procedure of qPCR and Western blot experiment was shown in Supporting Information S1. In addition, Western blotting was used to detect autophagy-related proteins (LC3B and Beclin-1) and NF-*κ*B pathway phosphorylation levels in THP-1 cells. The Western blotting detection steps can be found in Table S2.

### 2.4. Flow Cytometry Assay

The 2⁣′,7⁣′-dichlorodihydrofluorescein diacetate (DCFH-DA) probe kit (Beyotime, Nantong, China) was used to detect the concentration of ROS in THP-1 cells. Please refer to Supporting Information S2 for the detailed procedure of measuring intracellular ROS levels.

### 2.5. Cell Metabolomic Analysis

Cells were collected and centrifuged at 4°C to remove the culture medium. The cells were then rinsed twice with sterile phosphate-buffered saline to remove residual culture medium and serum. The abovementioned cell samples were transported on dry ice and delivered to Ekemo Tech Group Co., Ltd. in Shenzhen, China, for further untargeted metabolomic analysis. The steps for untargeted metabolomics analysis of the cells can be found in Supporting Information S3.

### 2.6. HE and Masson Staining of Mouse Kidney Tissue

Wax blocks (3 *μ*m) were continuously sliced into mouse kidney tissue and then used for HE and Masson staining after dewaxing and gradient ethanol dehydration. HE staining and Masson staining were done by the pathology department. The specimens were finally mounted with neutral resin and examined under a microscope and photographed. The percentage of blue collagen fibers (fibrotic areas) in Masson-stained sections of mouse kidneys is analyzed using ImageJ software to assess the degree of kidney fibrosis in mice.

### 2.7. Statistical Analysis

All data are denoted as the mean ± standard deviation (SD). SPSS 22.0 software (SPSS Inc, Chicago, IL) and GraphPad Prism 9 software (GraphPad Prism, San Diego, CA) were used for data analysis and mapping. The statistical significance was analyzed by ANOVA with post-Turkey's test. *p* < 0.05 was denoted as statistically significant.

## 3. Results

### 3.1. Mogrosides Inhibit High Glucose-Induced THP-1 Activation

**Figure 1:**
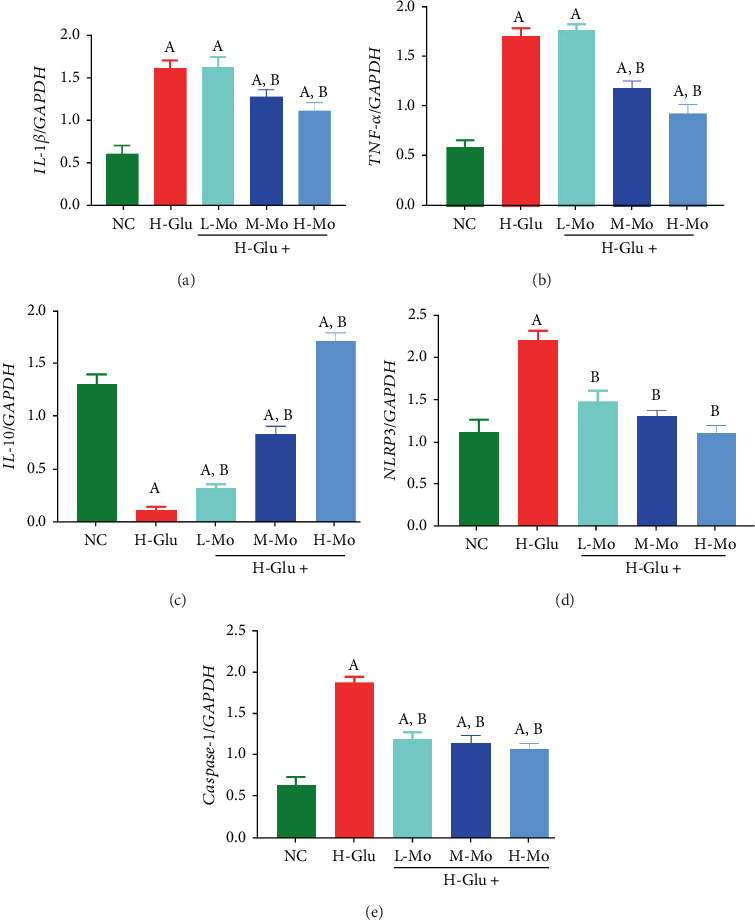
Mogrosides inhibits high-glucose-induced expressions of inflammatory cytokine mRNA in THP-1 cells, with significantly decreased expressions of the proinflammatory cytokines (a) *IL-1β*, (b) *TNF-α*, (d) *NLRP3*, and (e) *Caspase-1* (*p* < 0.05) and significantly increased expression of the anti-inflammatory cytokine (c) *IL-10*. (A) Compared with the NC group, *p* < 0.05. (B) Compared with the H-Glu group, *p* < 0.05.

In contrast to the NC group, THP-1 cells exposed to high glucose concentration for 12 h exhibited markedly elevated mRNA levels of *IL-1β*, *TNF-α*, *NLRP3*, and *Caspase-1* (*p* < 0.05), whereas *IL-10* mRNA expression showed a notable reduction (*p* < 0.05). When comparing with the H-Glu group, both M-Mo and H-Mo groups demonstrated substantially lower mRNA expressions of *IL-1β* and *TNF-α* (*p* < 0.05). The three concentrations of mogrosides could significantly inhibit the mRNA expressions of *NLRP3* and *Caspase-1* (*p* < 0.05) and increase the IL-10 expression (Figure 1).

### 3.2. Mogrosides Inhibit ROS Generation in High Glucose-Induced THP-1 Cells

**Figure 2:**
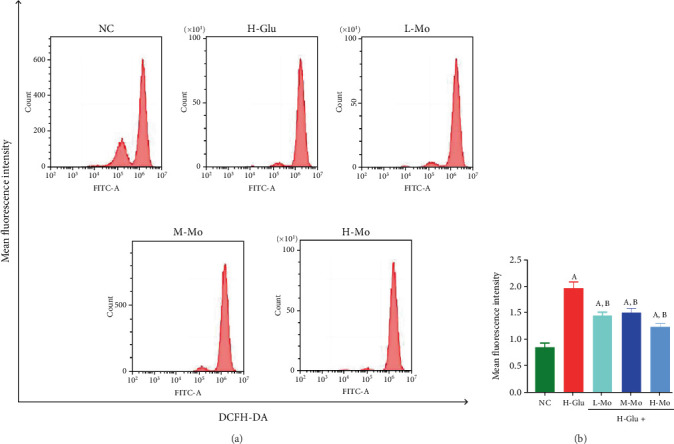
(a) Using flow cytometry to detect the generation of ROS in THP-1 cells. (b) The results showed that the three concentrations of mogrosides can significantly inhibit the generation of ROS in cells. (A) Compared with the NC group, *p* < 0.05. (B) Compared with the H-Glu group, *p* < 0.05.

In contrast to the NC group, the amount of ROS generated in THP-1 cells was notably elevated in H-Glu group (*p* < 0.05). Relative to the H-Glu group, mogrosides can significantly inhibit the intracellular ROS generation in THP-1 cells (*p* < 0.05) (Figure 2).

### 3.3. Mogrosides Inhibit High Glucose-Induced Activation of NLRP3 Inflammasome and Autophagy-Related Protein Expressions in THP-1 Cells

**Figure 3:**
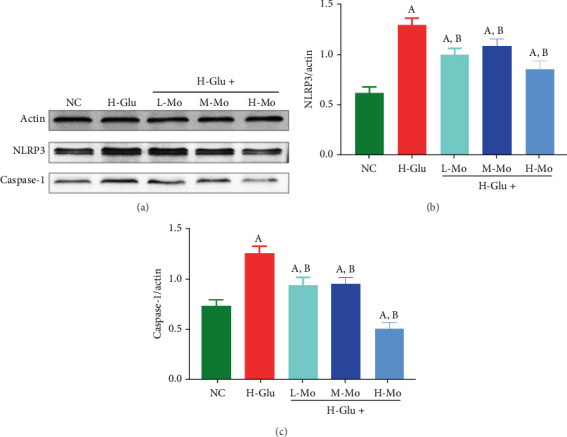
Using Western blotting assay to detect the expressions of (a) NLRP3 and Caspase-1 in THP-1 cells, all the three concentrations of mogrosides can significantly inhibit the expressions of (b) NLRP3 and (c) Caspase-1 induced by high concentrations of glucose in THP-1 cells. (A) Compared with the NC group, *p* < 0.05. (B) Compared with the H-Glu group, *p* < 0.05.

**Figure 4:**
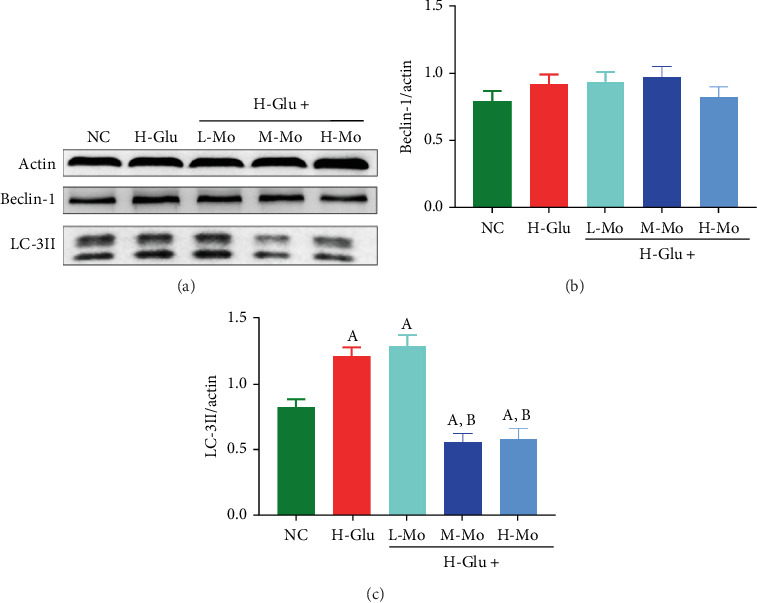
Using Western blotting assay to detect the expressions of autophagy-related proteins (a) Beclin-1 and LC-3II in THP-1 cells, medium and high concentrations of mogrosides can significantly inhibit the expression of autophagy-related protein LC3II induced by high glucose in (c) THP-1 cells, but there was no significant change in the expression of (b) Beclin-1. (A) Compared with the NC group, *p* < 0.05. (B) Compared with the H-Glu group, *p* < 0.05.

After treatment with high concentration glucose, the expressions of NLRP3 and Caspase-1 in THP-1 cells were notably elevated compared to the NC group (*p* < 0.05). However, when the high glucose-induced cells were treated with mogrosides, NLRP3 and Caspase-1 levels were notably reduced (*p* < 0.05), with high concentration of mogrosides having the best inhibitory effect (Figure 3). The LC3 II autophagy-related protein levels exhibited notable elevation in the H-Glu group versus the NC group (*p* < 0.05). In contrast, the administration of mogrosides at both medium and high doses demonstrated substantial suppression of LC3 II protein expression (*p* < 0.05) (Figure 4).

### 3.4. Mogrosides Inhibit the Phosphorylation Level of the NF-*κ*B Signaling Pathway in THP-1 Cells

**Figure 5:**
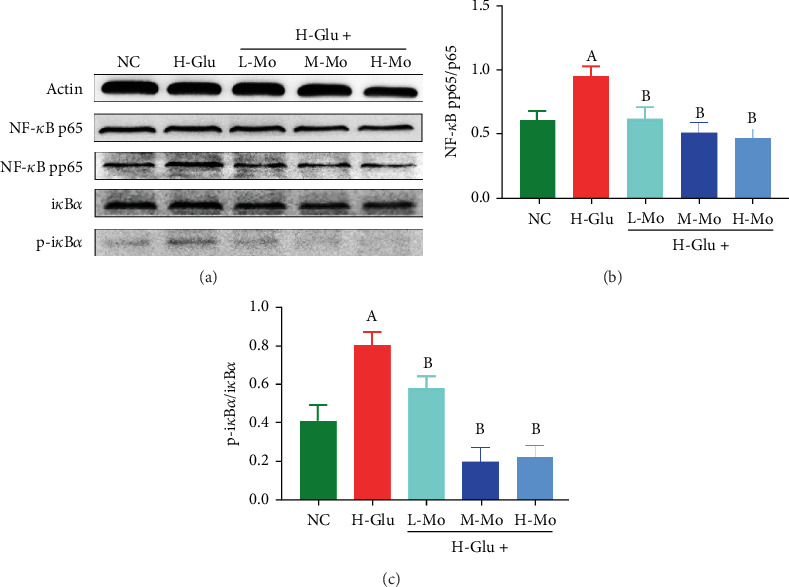
Using Western blotting assay to detect the phosphorylation levels of p65 protein and I*κ*B*α* protein in the (a) NF-*κ*B pathway of THP-1 cells, all the three concentrations of mogrosides can inhibit the phosphorylation of (b) p65 protein and (c) I*κ*B*α* protein. (A) Compared with the NC group, *p* < 0.05. (B) Compared with the H-Glu group, *p* < 0.05.

NF-*κ*B functions as a crucial transcription factor within cellular nuclei, serving essential functions in inflammatory reactions and immunological responses. The phosphorylation levels of p65 protein and I*κ*B*α* protein in the NF-*κ*B pathway are positively correlated with the degree of inflammatory response. In this study, after stimulation with high glucose concentration, NF-*κ*B pp65 and p-i*κ*B*α* levels in THP-1 cells were markedly elevated versus the NC group (*p* < 0.05), while treatment with the three concentrations of mogrosides could significantly reduce the phosphorylation levels of the NF-*κ*B signaling cascade in THP-1 cells (*p* < 0.05) (Figure 5).

### 3.5. Cell Metabolomic Analysis

**Figure 6:**
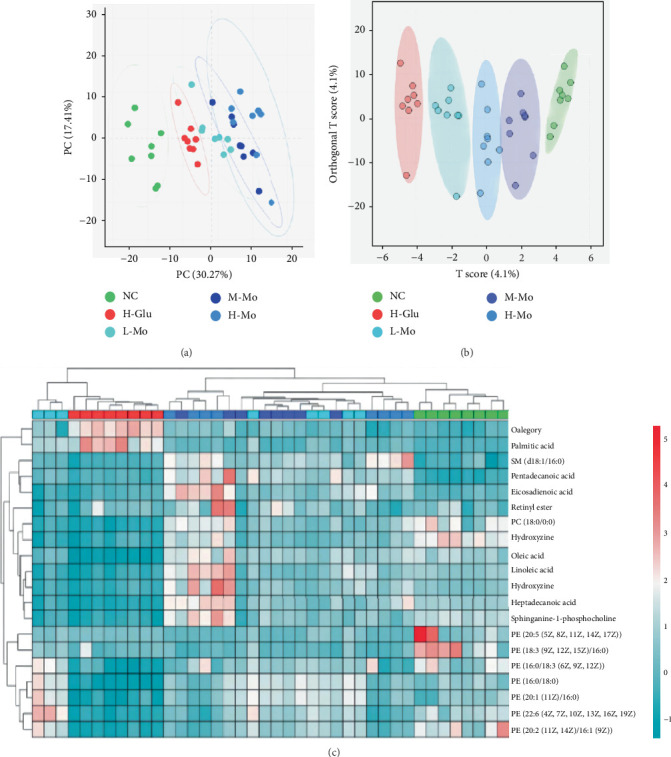
Metabolomics analysis results of THP-1 cells. The PCA results showed that the metabolomics of THP-1 cells after treatment with the three concentrations of mogrosides were significantly separated from the (a) H-Glu group. Further analysis using (b) OPLS-DA obtained a heat map of (c) differential metabolites in cells.

Progenesis QI software (Waters, Massachusetts, United States) was used to collect and analyze metabolic data (ESI^−^) from various cell samples. A total of 240 metabolites were detected in this investigation. The PCA results demonstrated that the samples from the NC group and the H-Glu group were completely separated and clustered, indicating that high glucose concentration can significantly affect the metabolism of THP-1 cells; the samples from the L-Mo group, M-Mo group, and H-Mo group were not clearly separated, suggesting that the differences in the metabolism of THP-1 cells exposed to varying levels of mogrosides were not significant (Figure 6a); however, the three concentrations of mogrosides groups were significantly separated from the H-Glu group, indicating that mogrosides can effectively reverse the metabolic disorder induced by high glucose in THP-1 cells. Further screening of different metabolites regulated by mogrosides was carried out through the OPLS-DA model and VIP > 1 and *p* < 0.05 rules (Figure 6b,c). The different metabolites can be found in Tables S3 and S4.

### 3.6. Metabolic Pathway Analysis

**Figure 7:**
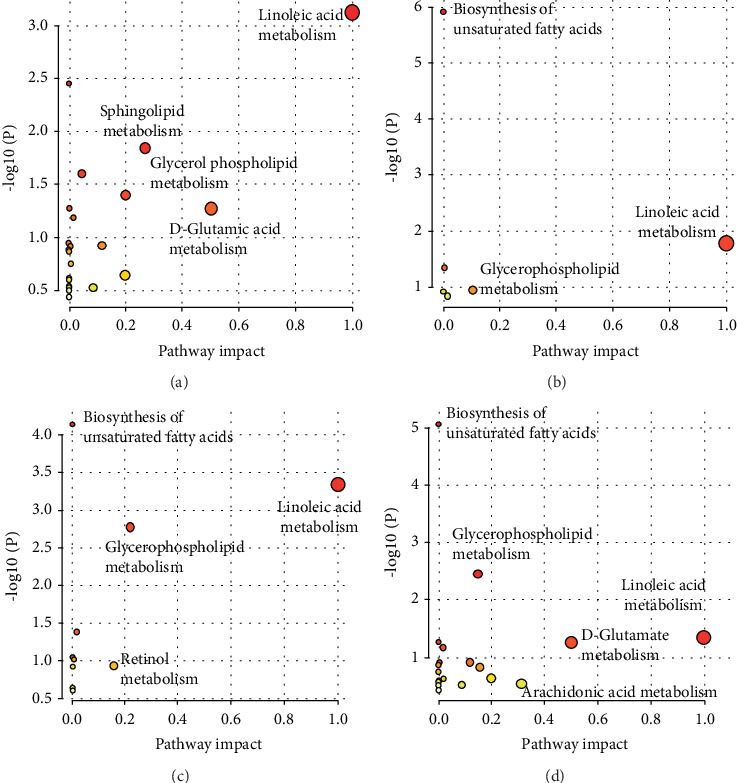
Enrichment analysis of metabolic pathways. (a) NC group and H-Glu group; (b) H-Glu group and L-Mo group; (c) H-Glu group and M-Mo group; (d) H-Glu group and H-Mo group.

The identified metabolites were entered into the MetaboAnalyst 5.0 platform to examine metabolic pathway alterations. The target value of pathway impact was set at 0.1, and any value greater than 0.1 was considered an affected metabolic pathway. The results showed that high glucose stimulation significantly affected multiple metabolic pathways in THP-1 cells, encompassing arachidonic acid metabolism, D-glutamate metabolism, sphingolipid metabolism, and glycerophospholipid metabolism; treatment with the three concentrations of mogroside significantly restored arachidonic acid metabolism, D-glutamate metabolism, and glycerophospholipid metabolism in THP-1 cells. These findings indicate that mogrosides potentially modulate the inflammatory reactions of high-glucose-induced THP-1 cells through the arachidonic acid metabolism, D-glutamate metabolism, and glycerophospholipid metabolism pathways (Figure 7).

### 3.7. Mogrosides Reduce Weight Gain and Abnormal Indicators Induced by a High-Fat Diet in Mice

**Figure 8:**
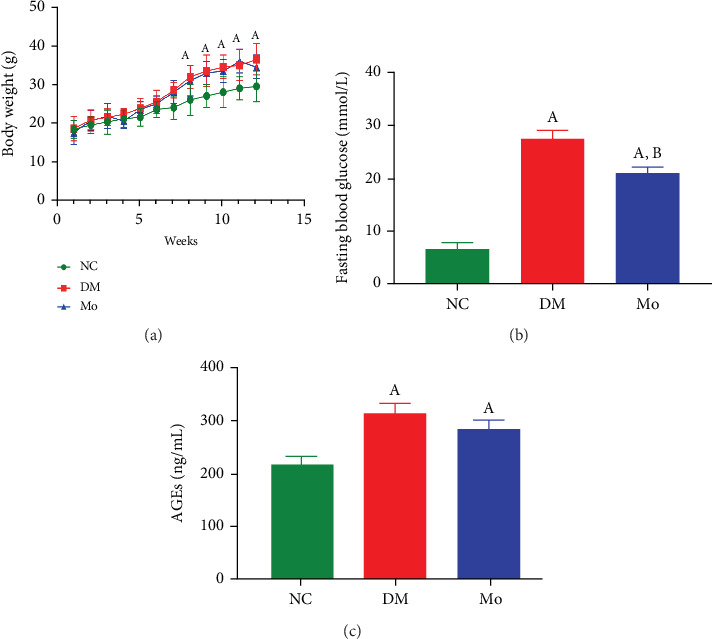
Changes in (a) body weight, (b) fasting blood glucose, and (c) AGE levels in mice. (A) Compared with the NC group, *p* < 0.05. (B) Compared with the DM group, *p* < 0.05.

During the experimental period spanning Weeks 1 through 12, alterations in mouse body weight and blood biochemical parameters were depicted in Figure 8. Starting at Week 8, the body weights of the DM and Mo groups were significantly increased compared with the NC groups (*p* < 0.05). There was no significant difference in body weight between the DM and Mo groups (*p* > 0.05).

At the end of the experiment, compared with the NC group, the levels of FBG in the DM and Mo groups were significantly increased (*p* < 0.05). Compared with the DM group, the FBG level of the Mo group was significantly decreased (*p* < 0.05). AGE levels in the DM and Mo groups were markedly elevated (*p* < 0.05), while there was no significant difference between the DM group and the Mo group (*p* > 0.05).

### 3.8. Mogrosides Inhibited the Expression of Proinflammatory Cytokines in Renal Tissues

**Figure 9:**
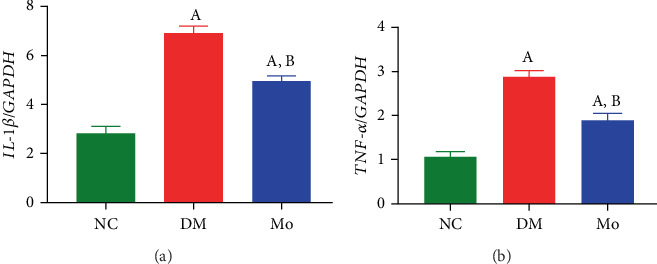
Using qPCR assay to detect the expressions of (a) IL-1*β* and (b) TNF-*α* in mouse kidney tissue. (A) Compared with the NC group, *p* < 0.05. (B) compared with the DM group, *p* < 0.05.

Relative to the NC group, significant elevations (*p* < 0.05) were detected in the mRNA expressions of proinflammatory cytokines IL-1*β* and TNF-*α* within kidney tissues of mice belonging to the DM and Mo groups. In comparison to the DM group, substantial reductions (*p* < 0.05) were detected in the mRNA expressions of IL-1*β* and TNF-*α* in the Mo group (Figure 9).

### 3.9. Mogroside Alleviates Kidney Damage and Fibrosis in Diabetic Mice

HE staining suggested that the renal tissue structure of the NC group mice was clear, the renal tubules were arranged in order, and the interstitial edema was not obvious. Renal tissue in the DM group showed significant expansion of glomerular volume, proliferation of mesangial cells and stroma, dilation of some renal tubules, and vacuolar degeneration of renal tubular epithelial cells. The above pathological changes in the Mo group mice were significantly alleviated after treatment with mogrosides.

**Figure 10:**
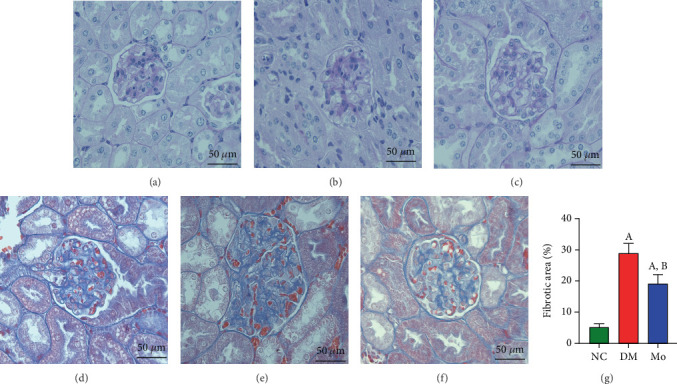
HE and Masson staining of mouse kidney tissue. (a–c) HE staining. (d–f) Masson staining. (a, d) NC group. (b, e) DM group. (c, f) Mo group. (g) Area of renal fibrosis in mice (%). (A) Compared with the NC group, *p* < 0.05. (B) Compared with the DM group, *p* < 0.05.

Masson staining results showed that the kidney tissue structure of mice in the NC group was normal, and there was no significant deposition of collagen fibers in the glomerular basement membrane and interstitium, while the glomerular basement membrane of mice in the DM group was significantly thickened, and a large number of collagen fibers were deposited in the renal interstitium, and the arrangement structure of the renal tubules was unclear. The collagen fiber deposition in the Mo group was markedly alleviated after mogroside administration (*p* < 0.05) (Figure 10).

## 4. Discussion

Our research found that oral mogrosides can provide numerous benefits to diabetic mice, including inhibiting macrophage activation, reducing inflammation and oxidative stress levels in diabetic mice, and alleviating kidney damage and fibrosis in the mice.

The pathogenesis of DKD is complex and involves changes in renal hemodynamics, oxidative stress, and metabolic disorders related to genetic background, hyperglycemia, and lipid metabolism abnormalities [22, 23]. However, the abnormal activation of macrophages serves as a critical mediator in the development of diabetic kidney injury [10, 24].

We found that high concentrations of glucose can significantly activate THP-1-derived macrophages, leading to a marked elevation in the expressions of the NLRP3 inflammasome, IL-1*β*, and TNF-*α*, while the expression of the anti-inflammatory cytokine IL-10 significantly decreases. We also noted a notable increase in inflammation levels in the kidneys of diabetic mice. After treating the cells and mice with mogrosides, the inflammation levels (such as the IL-1*β* and TNF-*α* expression) in both the cells and mice significantly decreased, with the inhibitory effect of high concentrations of mogrosides on macrophage inflammatory responses being the most pronounced. We believe that the inhibition of macrophage inflammatory responses by mogrosides is key to its therapeutic effects on diabetic kidneys.

It is known that the activation of NF-*κ*B is a key pathway for the progression of inflammation and fibrosis in diabetic nephropathy, and inhibiting NF-*κ*B activation can reduce the expressions of proinflammatory cytokines and prevent diabetes from progressing to kidney damage [25]. Other research results have shown that the NLRP3 inflammasome is involved in NF-*κ*B-mediated diabetic inflammation, and inhibiting the NF-*κ*B signaling pathway activation substantially reduces NLRP3 inflammasome activation in diabetic rats [26]. In the diabetic state, danger-associated molecular patterns (DAMPs), particularly elevated glucose levels, trigger the activation of the NF-*κ*B signaling pathway [27]. In the resting state, NF-*κ*B p65 protein binds specifically to I*κ*B kinase, but DAMPs can activate I*κ*B kinase and cause it to dissociate from the NF-*κ*B p65 protein, allowing NF-*κ*B p65 protein to enter the nucleus from the cytoplasm and ultimately activate the NF-*κ*B signaling pathway to induce inflammation [28].

We found that high glucose can significantly increase the generation of ROS in THP-1 cells. ROS is also a classical DAMP, and high concentrations of ROS can activate the NF-*κ*B signaling pathway and activate the NLRP3 inflammasome [29, 30]. However, when THP-1 cells were incubated with mogrosides, the generation of ROS in the cells was significantly reduced, and the expression of the anti-inflammatory cytokine IL-10 in the cells was significantly increased. We believe that mogrosides may inhibit macrophage activation by suppressing the activation of the NLRP3 inflammasome and the NF-*κ*B pathway.

The relationship between autophagy and diabetes is intricate, especially within the context of DKD. Autophagy, a conserved lysosomal degradation pathway, plays a vital role in preserving cellular homeostasis by recycling damaged organelles, such as mitochondria, and misfolded proteins under stress conditions like hyperglycemia, hypoxia, or oxidative stress [31]. However, recent evidence indicates that autophagy's role in DKD is context-dependent, with effects ranging from protective to harmful, influenced by the disease stage, cell type, and metabolic environment [32].

In the early stages of DKD, moderate activation of autophagy, indicated by increased LC3-II/Beclin-1 expression, helps reduce renal injury by clearing AGEs and dysfunctional mitochondria [33, 34]. This process alleviates oxidative stress and reduces NLRP3 inflammasome activation [35]. Our in vitro studies show that high glucose-induced autophagy in THP-1 macrophages may serve as a compensatory mechanism against high glucose-induced ROS, supporting findings that autophagy inhibition can worsen renal inflammation in diabetic models.

Conversely, prolonged autophagy activation in late-stage DKD can lead to adverse outcomes, such as podocyte loss and tubulointerstitial fibrosis [36]. Excessive autophagy might deplete vital cellular components or induce autophagic cell death [37, 38]. For example, podocyte-specific deletion of autophagy genes like Atg5 in podocytes exacerbates albuminuria, while unchecked autophagy accelerates renal aging [39, 40]. This dual nature highlights the importance of finely tuning autophagy in DKD treatment.

Our research indicates that mogrosides inhibit high glucose-induced autophagy in macrophages. Although this appears to contradict the typical view of autophagy as anti-inflammatory, it may reflect a regulation specific to cell type. We propose two possible, nonexclusive mechanisms. First, in maintaining redox balance, mogrosides might directly scavenge ROS, thereby reducing the autophagic demand triggered by oxidative stress [41]. Second, by preserving macrophage viability, mogrosides could prevent excessive immune cell loss without impairing pathogen clearance—an essential balance for diabetic patients susceptible to infections [42]. Future studies should explore whether mogrosides have temporally distinct effects on autophagy.

The metabolomic study suggests that the metabolism of arachidonic acid, D-glutamine, and glycerophospholipids in THP-1 cells may be potential mechanisms for mogrosides to regulate inflammatory responses. Glycerophospholipids are important components of cell membrane phospholipids, and about 60% of lipid molecules in the human body are glycerophospholipids [43, 44]. Phosphatidylcholine (PC) and phosphatidylethanolamine (PE) are the main metabolic products of glycerophospholipids [45]. PC is an essential phospholipid in the human body and plays a critical role in maintaining membrane fluidity and function [46, 47]. Previous studies have shown that PC helps maintain oxidative balance, inhibits inflammatory responses, and reduces liver and kidney damage [48]. In vitro studies have also shown that both PC and PE can reduce the phosphorylation of NF-*κ*B p65 protein in mouse macrophages (Raw 264.7 cells), inhibit inflammatory responses, and reduce the secretion of various proinflammatory cytokines [49]. In this study, we found that high glucose can regulate the glycerophospholipid metabolism pathway in THP-1 cells and reduce the content of PC and PE in cells, which may be a potential mechanism for high glucose to activate macrophage inflammatory responses. We also found that mogrosides can significantly increase the concentration of PE in THP-1 cells, indicating that mogrosides may inhibit macrophage inflammatory responses through the glycerophospholipid metabolism pathway.

Glutamine is the most abundant amino acid in the circulating and intracellular amino acid pools [50]. The decrease in glutamine concentration in the blood is closely related to immune dysfunction after trauma [51]. Mononuclear macrophages are an important component of the immune system, and glutamine is the main metabolic substrate of mononuclear macrophages. Glutamine provides energy for cellular metabolism through the glutamine catabolic pathway and provides purine, pyrimidine, and nucleotide biosynthetic precursors for the synthesis of DNA and mRNA [52]. The immune function of mononuclear macrophages depends on the concentration of glutamine. Inhibiting glutamine metabolism can induce macrophages to transform into M1 type and upregulate the expression of proinflammatory cytokines, while supplementing glutamine can promote macrophages to transform into M2 type and inhibit macrophage inflammatory response [53]. We found that high glucose can induce THP-1 cell inflammatory response, activate the NLRP3 inflammasome and NF-*κ*B signaling pathways, increase the expression of proinflammatory cytokines such as IL-1*β*, and decrease the content of glutamic acid in cells. However, after THP-1 cells were treated with mogrosides, the content of glutamic acid in cells increased, and the inflammatory response of cells was significantly inhibited. The above research results indicate that the D-glutamic acid metabolic pathway is also a potential target for mogroside.

In this study, we chose the ICR mouse strain primarily to prioritize the reproducibility and cost-effectiveness of the experiment for preliminary mechanistic exploration. Although our research confirmed the protective effect of mogrosides on diabetic kidney injury in ICR mice, we acknowledge that using only a single mouse strain presents some limitations in the experimental design. ICR mice lack the leptin receptor mutation present in db/db mice, which makes db/db mice a more prominent experimental model for obesity and hyperglycemia, closely resembling late-stage human T2DM. In the next phase of our research, we will use db/db mice and increase clinical trial studies to gain a more comprehensive understanding of the effects of mogrosides in treating diabetic kidney injury.

## 5. Conclusions

In summary, mogrosides can inhibit the inflammatory response induced by high glucose, and the common anti-inflammatory mechanism may be achieved through the NF-*κ*B/NLRP3/Caspase-1 axis. Further cellular metabolic research results show that the anti-inflammatory mechanism of mogrosides is related to their regulation of macrophage glutamine metabolism and glycerophospholipid metabolism pathways. Our research results suggest that supplementing mogrosides may help to inhibit chronic inflammation in diabetic patients and prevent complications such as DKD.

## Data Availability

The data that support the findings of this study are available from the corresponding authors upon reasonable request.

## Supporting Information

Supporting Information Additional supporting information can be found online in the Supporting Information section. Table S1: Nutrient composition table of 40% high-fat feed. Table S2: The primer sequences for qPCR assay. Table S3: Differential metabolites of THP-1 cells between the NC group and the H-Glu group. Table S4: Differential metabolites of THP-1 cells in the H-Glu group and three concentrations of mogroside groups (including the H-Mo group, the M-Mo group, and the L-Mo group). Supporting Information S1: qPCR assay and Western blotting assay. Supporting Information S2: Detecting intracellular ROS levels using flow cytometry. Supporting Information S3: Cell metabolomic analysis.
