# Supplementary material for: Mogrosides Protect Against Diabetic Kidney Injury via Inhibiting Macrophage Activation in a Mouse Model
Source: J Diabetes Res. 2025 Jun 24;2025:5291562. doi: 10.1155/jdr/5291562 (PMC12213039; doi:10.1155/jdr/5291562)
Supplement: Supporting Information — Additional supporting information can be found online in the Supporting Information section. Table S1: Nutrient composition table of 40% high-fat feed. Table S2: The primer sequences for qPCR assay. Table S3: Differential metabolites of THP-1 cells between the NC group and the H-Glu group. Table S4: Differential metabolites of THP-1 cells in the H-Glu group and three concentrations of mogroside groups (including the H-Mo group, the M-Mo group, and the L-Mo group). Supporting Information S1: qPCR assay and Western blotting assay. Supporting Information S2: Detecting intracellular ROS levels using flow cytometry. Supporting Information S3: Cell metabolomic analysis. [file 5291562.f1.doc]

**Supplement**

Table S1 Nutrient composition table of 40% high-fat feed

| Product | gm | kcal(%) |
| --- | --- | --- |
| Protein | 23 | 20 |
| Carbohydrate | 46 | 40 |
| Fat | 20 | 40 |
| Total |  | 100 |
| kcal/gm | 4.55 | - |

#### Table S2. The primer sequences for qPCR assay

| Gene | Primer sequences（5'→3'） |
| --- | --- |
| *Mouse-GAPDH* | F: CATCACTGCCACCCAGAAGACTG  R: ATGCCAGTGAGCTTCCCGTTCAG |
| *Mouse-IL-1β* | F:GCAACTGTTCCTGAACTCAACT  R:ATCTTTTGGGGTCCGTCAACT |
| *Mouse-TNF-α* | F:CCCTCACACTCAGATCATCTTCT  R:GCTACGTGGGCTACAG |
| *THP-1_GAPDH* | F: CATCACTGCCACCCAGAAGACTG  R: ATGCCAGTGAGCTTCCCGTTCAG |
| *THP-1_IL-1β* | F: CCTGTCCTGCGTGTTGAAAGA  R: GGGAACTGGGCAGACTCAAA |
| *THP-1_TNF-α* | F: AATGGCGTGGAGCTGAGA  R: TGGCAGAGAGGAGGTTGAC |
| *THP-1_NLRP3* | F: AACAGCCACCTCACTTCCAG  R: CCAACCACAATCTCCGAATG |
| *THP-1_Caspase-1* | F: GCACAAGACCTCTGACAGCA  R: TTGGGCAGTTCTTGGTATTC |
| *THP-1_IL-10* | F: TCTCCGAGATGCCTTCAGCAGA  R: TCAGACAAGGCTTGGCAACCCA |

Table S3: Differential metabolites of THP-1 cells between the NC group and H-Glu group

| No | Compounds | Formula | HMDB | R/ T (min) | m/z | Change |
| --- | --- | --- | --- | --- | --- | --- |
| 1 | L-Glutamic acid | C5H9NO4 | HMDB00148 | 344.06 | 1.11 | ↓ |
| 2 | PC(18:3(9Z,12Z,15Z)/18:0) | C44H82NO8P | HMDB08201 | 806.57 | 23.34 | ↓ |
| 3 | Oxidized glutathione | C20H32N6O12S2 | HMDB03337 | 613.16 | 1.82 | ↑ |
| 4 | Prostaglandin F1a (PGF1a) | C20H36O5 | HMDB02685 | 730.54 | 21.89 | ↑ |
| 5 | N-Methyltryptamine | C11H14N2 | HMDB04370 | 387.19 | 5.36 | ↑ |
| 6 | Elaidic acid | C18H34O2 | HMDB00573 | 356.35 | 5.52 | ↓ |
| 7 | Androsterone | C19H30O2 | HMDB00031 | 291.23 | 7.77 | ↑ |
| 8 | Palmitic acid | C16H32O2 | HMDB00220 | 272.26 | 4.88 | ↑ |
| 9 | 13S-hydroxyoctadecadienoic acid | C18H32O3 | HMDB04667 | 314.27 | 4.92 | ↑ |
| 10 | Floionolic acid | C18H36O5 | HMDB34295 | 331.25 | 7.72 | ↑ |
| 11 | Dolichyl b-D-glucosyl phosphate | C21H39O9P | HMDB01054 | 465.23 | 7.52 | ↓ |
| 12 | PE(22:6(4Z,7Z,10Z,13Z,16Z,19Z) | C49H78NO8P | HMDB09702 | 884.54 | 16.23 | ↓ |
| 13 | Eicosadienoic acid | C19H36O2 | HMDB05060 | 295.27 | 13.85 | ↑ |
| 14 | Hexyl octanoate | C20H36O2 | HMDB36216 | 307.27 | 13.61 | ↑ |
| 15 | CDP-DG(16:0/16:0) | C14H28O2 | HMDB06968 | 227.20 | 13.85 | ↓ |
| 16 | Retinol acetate | C44H81N3O15P2 | HMDB35185 | 952.51 | 16.29 | ↑ |
| 17 | Tetrahydrocorticosterone | C22H32O2 | HMDB00268 | 327.23 | 12.32 | ↑ |
| 18 | Oleic acid | C21H34O4 | HMDB00207 | 349.24 | 13.44 | ↑ |
| 19 | Pteroside A | C18H34O2 | HMDB30758 | 281.25 | 13.44 | ↑ |
| 20 | Eremopetasin sulfoxide | C21H30O8 | HMDB32003 | 455.19 | 12.86 | ↑ |
| 21 | Bortezomib | C19H26O4S | HMDB14334 | 395.15 | 13.37 | ↑ |
| 22 | Myristic acid | C19H25BN4O4 | HMDB00806 | 429.20 | 12.69 | ↑ |
| 23 | Sciadonic acid | C14H28O2 | HMDB31058 | 227.20 | 12.45 | ↑ |
| 24 | (S)-[8]-Gingerol | C20H34O2 | HMDB33615 | 305.25 | 13.10 | ↑ |
| 25 | PE(20:2(11Z,14Z)/16:1(9Z)) | C19H30O4 | HMDB09287 | 321.21 | 12.62 | ↑ |
| 26 | Heptadecanoic acid | C41H76NO8P | HMDB02259 | 786.53 | 11.36 | ↑ |
| 27 | PE(20:1(11Z)/16:0) | C17H34O2 | HMDB09253 | 269.25 | 13.85 | ↓ |
| 28 | Hydroxyzine | C41H80NO8P | HMDB14697 | 790.59 | 16.26 | ↑ |
| 29 | Palmitic acid | C21H27ClN2O2 | HMDB00220 | 355.16 | 13.37 | ↑ |
| 30 | Pentadecanoic acid | C15H30O2 | HMDB00826 | 241.22 | 12.89 | ↑ |
| 31 | Linoleic acid | C18H32O2 | HMDB00673 | 279.23 | 12.69 | ↓ |
| 32 | PE(20:5(5Z,8Z,11Z,14Z,17Z)) | C43H74NO8P | HMDB09455 | 808.51 | 16.23 | ↓ |
| 33 | SM(d18:1/16:0) | C47H95N2O6P | HMDB10169 | 859.69 | 16.29 | ↓ |

Table S4: Differential metabolites of THP-1 cells in the H-Glu group and three concentrations of mogroside groups (including H-Mo group, M-Mo group, and L-Mo group)

| No | Compounds | Formula | HMDB | R/ T (min) | m/z | L-Mo | M-Mo | H-Mo |
| --- | --- | --- | --- | --- | --- | --- | --- | --- |
| 1 | Stearic acid | C18H36O2 | HMDB00827 | 14.39 | 283.26 | ↑ | ↑ | ↑ |
| 2 | Sphinganine-1-phosphocholine | C23H51N2O5P | HMDB12082 | 13.58 | 447.33 | ↑ | ↑ | ↑ |
| 3 | SM(d18:1/16:0) | C47H95N2O6P | HMDB10169 | 16.29 | 859.69 | ↑ | ↑ | ↑ |
| 4 | Retinyl ester | C20H30O2 | HMDB03598 | 12.69 | 347.22 | ↑ | ↑ | ↑ |
| 5 | PI(18:1(9Z)/20:3(8Z,11Z,14Z)) | C47H83O13P | HMDB09843 | 16.23 | 885.55 | ↑ | ↑ | ↑ |
| 6 | PI(18:0/18:2(9Z,12Z)) | C45H83O13P | HMDB09809 | 16.23 | 861.55 | ↑ | ↑ | ↑ |
| 7 | PI(16:0/18:1(9Z)) | C43H81O13P | HMDB09783 | 16.23 | 835.53 | ↑ | ↑ | ↑ |
| 8 | Pentadecanoic acid | C15H30O2 | HMDB00826 | 12.89 | 241.22 | ↑ | ↑ | ↓ |
| 9 | PE(20:5(5Z,8Z,11Z,14Z,17Z)) | C43H74NO8P | HMDB09455 | 16.23 | 808.51 | ↑ | ↑ | ↑ |
| 10 | PE(18:3(9Z,12Z,15Z)/16:0) | C39H72NO8P | HMDB09154 | 11.36 | 758.50 | ↑ | ↑ | ↑ |
| 11 | PE(16:0/18:3(6Z,9Z,12Z)) | C39H72NO8P | HMDB08929 | 16.26 | 758.50 | ↑ | ↑ | ↑ |
| 12 | PE(16:0/18:0) | C39H78NO8P | HMDB08925 | 16.26 | 700.53 | ↑ | ↑ | ↑ |
| 13 | Palmitic acid | C16H32O2 | HMDB00220 | 13.37 | 255.23 | ↓ | ↓ | ↓ |
| 14 | Oleic acid | C18H34O2 | HMDB00207 | 13.44 | 281.25 | ↓ | ↓ | ↓ |
| 15 | Palmitoleic acid | C16H30O2 | HMDB03229 | 13.85 | 253.22 | ↓ | ↓ | ↓ |
| 16 | Linoleic acid | C18H32O2 | HMDB00673 | 12.69 | 279.23 | ↑ | ↑ | ↑ |
| 17 | Hydroxyzine | C21H27ClN2O2 | HMDB14697 | 13.37 | 355.16 | ↑ | ↑ | ↑ |
| 18 | Heptadecanoic acid | C17H34O2 | HMDB02259 | 13.85 | 269.25 | ↑ | ↑ | ↑ |
| 19 | Eicosadienoic acid | C20H36O2 | HMDB05060 | 13.61 | 307.27 | ↑ | ↑ | ↑ |
| 20 | Cyclohexaneundecanoic acid | C17H32O2 | HMDB30997 | 13.85 | 267.23 | ↑ | ↑ | ↑ |
| 21 | PC(18:0/0:0) | C26H54NO7P | HMDB10384 | 11.84 | 568.36 | ↑ | ↑ | ↑ |
| 22 | UDP-N-acetylmuraminate | C17H27N3O17P2 | HMDB11720 | 1.11 | 344.06 | ↓ | ↓ | ↓ |
| 23 | TXB2 | C20H34O6 | HMDB03252 | 11.81 | 390.36 | ↓ | ↓ | ↓ |
| 24 | L-Glutamic acid | C5H9NO4 | HMDB00148 | 9.16 | 365.27 | ↑ | ↑ | ↑ |
| 25 | PGF1a | C20H36O5 | HMDB02685 | 6.96 | 482.36 | ↑ | ↑ | ↑ |

**S1: QPCR assay and Western Blotting Assay**

Total RNA was extracted using the RNA-easy Isolation Reagent kit (Vazyme, Nanjing, China), and cDNA was synthesized using the HiScript III 1^st^ Strand cDNA Synthesis Kit (+gDNA Wiper) kit. The qPCR reactions were performed using the AceQ Universal SYBR qPCR Master Mix kit to detect inflammation-related genes. The primer sequences for THP-1 cell inflammation-related factors are shown in Table S2 and were synthesized by GENEWIZ (Suzhou, China).

THP-1 cells were sonicated after adding an appropriate amount of RAPI lysis buffer (Beyotime, Nanjing, China), and the supernatant was collected after centrifugation. The protein concentration in the sample was determined using a BCA protein quantification assay kit (Beyotime, Nanjing, China). An appropriate amount of 2×SDS-PAGE loading buffer was added to the protein sample, and the sample was boiled at 100 ℃ to denature the proteins. The SDS-PAGE electrophoresis was then performed, and the proteins were transferred to a PVDF membrane. After transfer, the PVDF membrane was blocked with 5% bovine serum albumin and incubated with an appropriate amount of antibody overnight at 4 ℃. The antibodies used were provided from Proteintech Group, Lnc. (Wuhan, China), including NF-κB p65 (1:1000), NF-κB pp65 (1:500), iκBα (1:1000), p-iκBα (1:500), LC3B (1:500), and Beclin-1 (1:1000). Actin, as an internal reference antibody (1:10,000), was provided by BOSTER Biological Technology company (Wuhan, China).

After washing the PVDF membrane with TBST, an appropriate amount of secondary antibody labeled with horseradish peroxidase was added and incubated at room temperature for 1 hour. After washing away the unbound antibodies with TBST, an appropriate amount of SuperSignal West Atto (Thermo Fisher Scientific, Waltham, USA) was added for visualization, and the protein bands were analyzed and the band intensity was calculated.

**S2: Detecting Intracellular ROS Levels using Flow Cytometry**

The specific operating steps are as follows: THP-1 cells were collected and washed with sterile phosphate-buffer solution to remove residual culture medium and fetal bovine serum. DCFH-DA probe was diluted to a final concentration of 10 μmol/L with serum-free culture medium. The collected cells were added to the DCFH-DA probe solution and incubated at 37℃ for 20 min. The cells were washed three times with serum-free culture medium to remove unbound DCFH-DA probe. Finally, the cells were resuspended in sterile phosphate buffer solution for flow cytometry detection.

**S3: Cell metabolomics analysis**

Cells were collected and centrifuged at 4°C to remove the culture medium. The cells were then washed twice with sterile phosphate-buffered saline to remove residual culture medium and serum. The cells were resuspended in 2 mL of 80% methanol-water (v/v) and placed at -80°C for 20 min. The cells were then lysed using a cell ultrasonic disrupter, and the supernatant was collected after centrifugation and dried using nitrogen gas. Before detection, 200 µL of 50% acetonitrile-water (v/v) was added to each sample and re-dissolved for metabolomics detection.

The UPLC/Q-TOF MS system (Waters, Massachusetts, USA) was used, with an Acquity UPLC BEH C_18_ column (2.1 mm × 50 mm, 1.7 μm), an injection volume of 5 μL, a flow rate of 0.4 mL/min, and a column temperature of 45°C. The mobile phase A was water + 0.1% formic acid, and the mobile phase B was acetonitrile + 0.1% formic acid. The gradient elution program started with 5% B, increased to 95% B at 10 min, and then decreased to 5% B at 12 min. ESI ion source was used, and both positive and negative ion modes were used for full ion detection.

The raw mass spectrometry data obtained by UPLC/Q-TOF MS were imported into the MarkerLynx software package (Waters, Massachusetts, USA) for peak detection and matching, generating a three-dimensional data matrix containing retention time, mass-to-charge ratio, and peak area. The matrix was saved as an Excel file and imported into SIMCA-P software (UmetricSAB, Ume, Sweden) for pattern recognition. The variables with a variable important in projection (VIP) value greater than 1 were selected as the differential variables. The structure identification of differential metabolites was carried out by initially confirming the possible molecular formula based on the accurate molecular weight in Masslynx, and then confirming the structural formula of differential metabolites through databases such as Metlin, HMDB, Massbank, and ChemSpider.
